# Therapeutic effects of human adipose tissue-derived stem cell (hADSC) transplantation on experimental autoimmune encephalomyelitis (EAE) mice

**DOI:** 10.1038/srep42695

**Published:** 2017-02-15

**Authors:** Jia Li, Ying Chen, Zhibo Chen, Yuanyuan Huang, Dehao Yang, Zhongqian Su, Yiyun Weng, Xiang Li, Xu Zhang

**Affiliations:** 1 Department of Neurology, the First Affiliated Hospital of Wenzhou Medical University, Wenzhou 32500, Zhejiang, China

## Abstract

This study is to investigate the therapeutic effects of human adipose tissue-derived stem cell (hADSC) transplantation on experimental autoimmune encephalomyelitis (EAE) in mice. EAE mouse model was established by MOG35-55 immunization. Body weight and neurological function were assessed. H&E and LFB staining was performed to evaluate histopathological changes. Flow cytometry was used to detect Th17 and Treg cells. ELISA and real-time PCR were performed to determine transcription factor and pro-inflammatory cytokine levels. Transplantation of hADSCs significantly alleviated the body weight loss and neurological function impairment of EAE mice. Inflammatory cell infiltration and demyelination were significantly increased, which were relieved by hADSC transplantation. Moreover, the Th17 cells and the ROR-γt mRNA level were significantly elevated, while the Treg cells and the Foxp3 mRNA level were significantly declined, resulting in significantly increased Th17/Treg ratio. This was reversed by the transplantation of hADSCs. Furthermore, serum levels of IL-17A, IL-6, IL-23, and TGF-β, were significantly increased, which could be influenced by the hADSC transplantation. Transplantation of hADSCs alleviates the neurological function impairment and histological changes, and reduces the inflammatory cell infiltration and demyelination in EAE mice, which might be associated with the regulation of Th17/Treg balance.

Multiple sclerosis (MS) is an inflammatory, demyelinating, neurodegenerative disorder in the central nervous system (CNS), which is induced by the repeated activation of the autoimmune system. MS is one of the most common progressive neurological disorders in young people, leading to even higher social and economic costs than stroke and Alzheimer’s disease^1^. However, since the first report of MS in 1868, the exact disease pathogenesis has not yet been fully elucidated. Numerous studies indicate that the CD4^+^ T cells are major effector cells in the pathogenesis of MS^2^, and the imbalance between Th1/Th2 cells has also been recognized as a key player in the disease development^3^. Moreover, recent investigation suggests that, the Th17/Treg imbalance plays a key role in the pathogenesis and development of MS^4^. As a CD4^+^ T cell subset, Th17 cells are negative immunoregulatory cells involved in the immune tolerance, which secretes unique pro-inflammatory cytokines mediating inflammatory responses. Pro-inflammatory cytokines (i.e., IL-17A, IL-17F, IL-21, and IL-22) would attack the myelin and axons, leading to inflammatory injuries in MS, which could be inhibited by the regulatory T (Treg) cells^5^.

At present, the clinical treatments of MS are mainly focusing on the disease symptoms, only delaying the disease progression with various hormones and IFN-β^1^,^2^. However, ideal therapeutic strategies for MS should be able to interfere with the autoimmune process, prevent the spread of inflammation, repair the demyelinating injuries, and continuously adjust the pathogenic factors in the body. Along with the development of biotechnology and cytology in recent years, cellular immunotherapy has been attracting more and more attention concerning its clinical application for the disease treatment. Mesenchymal stem cells (MSCs), characterized by self-renewal capacity and pluripotency, exist in a variety of tissues and are able to regulate the inflammation and immune responses^6^. The efficiency and safety of bone marrow-derived mesenchymal stem cells (BMSCs) have been demonstrated in the clinical treatment of MS^7^. On the other hand, compared with BMSCs, the adipose tissue-derived stem cells (ADSCs) could be more easily obtained from autologous tissues, which are also featured with strong cloning capacity *in vitro* and low immunogenicity, without ethical concerns^8^,^9^,^10^.

Experimental autoimmune encephalomyelitis (EAE) shares similar pathological characteristics and clinical manifestations with human MS, which has been recognized as the ideal animal model for the disease simulation. MSCs could exert immunomodulatory and anti-inflammatory effects in various tissues^11^. BMSCs have also been shown to regulate immunity, relieve nerve damages, and inverse demyelination in the EAE models^12^. Previous studies have also shown that, BMSCs could induce the immune tolerance and T cell anergy, then alleviating the EAE symptoms^12^. On the other hand, when labeled with GFP, MSCs have been shown to be able to migrate to the lesion sites in the CNS, while the majority of MSCs accumulate and exert functions in the peripheral lymphoid organs^12^,^13^. MSCs could promote the expression of Foxp3 transcriptional factor in the Treg cells, playing a role in the immune regulation^14^,^15^,^16^. Moreover, a previous study has also demonstrated that, MSCs could inhibit the differentiation of Th17 cells via secreting prostaglandin E2 (PGE2), without influencing the differentiation of Treg cells^17^. However, no consensus has been reached on the regulatory effects of MSCs on the Th17/Treg axis. As for ADSCs, limited investigation has been reported for the effects of human ADSCs (hADSCs) on the differentiation of Th cells and related mechanisms.

Our previous study primarily demonstrates that hADSCs could inhibit the differentiation of Th17 cells via specifically secreting leukemia inhibitory factor (LIF)^18^. In this study, the therapeutic effects of hADSC transplantation on the EAE models, as well as the effects of hADSCs on the differentiation of Th17/Treg cells, were investigated and analyzed.

## Materials and Methods

### Isolation, culture, and identification of hADSCs

Human adipose tissue (5–10 g) was extracted from 5 healthy Chinese young females (aging from 22–35) under sterile condition, who received liposuction surgery at the Department of Plastic Surgery, the First Affiliated Hospital of Wenzhou Medical University. Subjects with any potentially confounding conditions, including diabetes, hypertension, hyperlipidemia, or autoimmune diseases were excluded from the study^19^. Prior written and informed consent were obtained from every patient and the study was approved by the ethics review board of the First Affiliated Hospital of Wenzhou Medical University. We confirm that all methods were performed in accordance with the ethical guidelines of the First Affiliated Hospital of Wenzhou Medical University. We confirm that informed consent was obtained from all subjects. These patients were free from autoimmune diseases, infectious diseases, and/or other major diseases. The tissue was cut into pieces, washed, and digested. After centrifugation, the cell precipitation was treated with 3 mL erythrocyte lysate for 3–5 min, and then centrifuged again. The cells were re-suspended and cultured with the LG-DMEM medium (HyClone, Logan, Utah, USA), supplemented with 1% penicillin and streptomycin (Solarbio, Beijing, China), in a 37 °C, 5% CO_2_ incubator for 3 d. When 70–90% confluence was reached, these cells were digested, centrifuged, and re-suspended to obtain amplified and purified hADSCs.

The hADSCs from the 3–5 passages were harvested, digested, and centrifuged. After washing, these cells were diluted into 5 × 10^6^/600 μL single-cell suspension in EP tubes (100 μL in each tube). The cells were then treated with mouse anti-human CD44-FITC, CD105-APC, CD29-FITC, CD73-PE, CD31-PE, HLA-DR-APC, CD13-FITC, CD34-APC, and CD49d-PE (all from eBioscience, San Diego, CA, USA), respectively, for 30 min, followed by the detection with flow cytometry. For the control group, the cells were incubated with FITC-IgG1, APC-IgG1, and PE-IgG1 (all from eBioscience), respectively.

### Animal modeling, grouping, and cell transplantation

Female C57BL/6 mice (SPF level), 6–8-week old, were purchased from SLAR Laboratory Animal, Shanghai, China (license number: SCXK Shanghai 2007-0005). All animal experiments were conducted according to the ethical guidelines of the First Affiliated Hospital of Wenzhou Medical University. We confirm that all methods were performed in accordance with the ethical guidelines of the First Affiliated Hospital of Wenzhou Medical University. For animal modeling, the immunogen/CFA emulsion was first prepared under sterile condition. These mice were divided into the following groups (n = 15): 1) the adjuvant control group, in which the mice were subcutaneously injected with the PBS/CFA emulsion; 2) the EAE model (EAE + PBS) group, in which the mice were immunized with the MOG35-55/CFA emulsion, and 14 days later, subjected to the injection of PBS via tail vein; and 3) the hADSC transplantation (EAE + hADSCs) group, in which the EAE mice were transplanted with hADSCs via tail vein.

For the cell transplantation, the hADSCs (3–5 passages) were harvested and counted to obtain the cell suspension at the density of 1 × 10^7^ cells/mL. At day 14 after immunization, for the hADSC transplantation group, each mouse was injected with 200 μL hADSC suspension via the tail vein. For the EAE model group, the mice were injected with 200 μL PBS instead.

### Body weight recording and neurological function assessment

From day 0 to day 30 after immunization, animal body weight was recorded, and the neurological function was assessed, according to the 5-point scoring criteria as previously published (scores of ≥1 suggested the successful establishment of the EAE models)^20^.

### Histological staining

At day 30 after immunization, the spinal cord samples were extracted. The tissue was cut into sections, which were subjected to the H&E staining and the Luxol Fast Blue (LFB; 0.1%) staining. The sections were observed under microscope, and the number of inflammatory cells and the percentage of the demyelination area to the whole area were counted and calculated with the Image-Pro Plus software.

### Flow cytometry

Th17 and Treg cells were detected with flow cytometry. Fresh spleen tissue was obtained under sterile condition, and single lymphocyte suspension (2–5 × 10^6^ cells/mL) was prepared. After treatment with stimulating or inhibiting agents, these cells were incubated with antibodies against Th17 and Treg cells (rat anti-mouse CD4-FITC, IL17A-PE, CD25-APC, and Foxp3-PE; all from eBioscience), respectively. Fluorescence was detected with a flow cytometer (FACSCalibur; BD, Franklin Lakes, NJ, USA).

### Quantitative real-time PCR

Total RNA were extracted from the single lymphocyte suspension. The cDNA was obtained using the reverse transcription kit (Thermo Fisher Scientific, Waltham, MA, USA). Quantitative real-time PCR was performed with SYBR Green (Takara, Madison, WI, USA) on the Applied Biosystems 7500 machine (Applied Biosystems, Foster City, CA, USA). Primer sequences were as follows: ROR, forward 5′-GAACCAGAACAGGGTCCAGA-3′ and reverse 5′-TCGGAAGGACTTGCAGACAT-3′; Foxp3, forward 5′-ACTCGCATGTTCGCCTACTT-3′ and reverse 5′-GTCCACACTGCTCCCTTCTC-3′; and IL-17, forward 5′-TCCCTCTGTGATCTGGGAAG-3′ and reverse 5′-CTCGACCCTGAAAGTGAAG-3′. The reaction conditions consisted of 50 °C for 2 min, 95 °C for 30 s, followed by 95 °C for 5 s, 62 °C for 34 s, for totally 40 cycles. Relative expression levels of target genes were calculated.

### Enzyme-linked immunosorbent assay (ELISA)

Blood was collected by the orbital exsanguinations from each mouse, and serum sample was then obtained. The contents of IL-17A, IL-6, IL-23, and TGF-β in serum were assessed with commercially available ELISA kits (eBioscience), respectively, according to the manufacturer’s instructions.

### Immunofluorescence

The hADSCs were labeled with PKH67 (Sigma-Aldrich) before injection^21^, according to the manufacturer’s instructions. Mice injected with unlabeled BMSCs were used as control. PKH671 cells were tracked exclusively inside the spleen, brain, and liver on day 8 after ADSC injection. Samples were cut into 5-mm sections on a cryostat, and observed by fluorescent microscopy (Olympus, Japan) after stained with DAPI (Sigma-Aldrich) for 5 min. Then the sections were mounted with glycerol buffered with PBS.

### Statistical analysis

Data were expressed as mean ± SD. SPSS 22.0 software was used for statistical analysis. For data with normality and homogeneity of variance, group comparison was performed by ANOVA, with the LSD test for multiple comparisons; if not, the Kruskal-Wallis test was conducted. *P* < 0.05 was considered as statistically significant.

## Results

### Culture and identification of hADSCs

**Figure 1:**
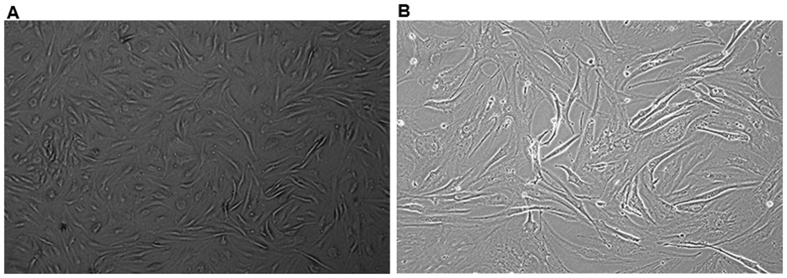
Morphological observation of cultured hADSCs. (**A**) The 2^nd^ passage cells (100×). (**B**) The 3^rd^ passage cells (400×).

Our results indicated that, at the 2–3 passages, the purity of our cultured hADSCs would be over 95%. Microscopic observation showed that, the adherent cells exhibited fibroblast-like morphology (Fig. 1). Results from flow cytometry showed that, for these cultured cells, the expression rates of CD13, CD29, CD44, CD73, and CD105 were all above 95%, while the expression rates of CD31, CD34, and HLA-DR were all below 3% (data not shown). Moreover, CD49d was partially expressed in these cultured cells (data not shown). These results suggest that the cultured cells are actually hADSCs, which are suitable for the cell transplantation and following investigation.

### Effects of hADSC transplantation on body weight and neurological function of EAE mice

**Figure 2:**
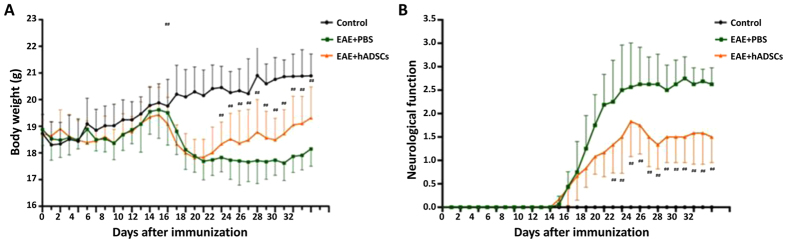
Effects of hADSC transplantation on the body weight and neurological function of the EAE mice. EAE mouse model was established by the immunization with the MOG35-55/CFA emulsion, and 14 days later, these mice were transplanted with hADSCs via tail vein. Animal body weight (**A**) and neurological function (**B**) of the mice in the control, EAE model, and hADSC transplantation groups were recorded and assessed since modeling. Compared with the EAE model group, ^##^*P* < 0.01.

The EAE mouse model was established by MOG35-55 immunization, and these EAE mice were transplanted with the above hADSCs. The body weight and neurological function were assessed. Our results showed that, compared with the control group, the averaged body weight was dramatically declined in the EAE model mice. However, the transplantation of hADSCs drastically elevated the body weight of these EAE mice (Fig. 2A). On the other hand, for the neurological function assessment (the score for control was 0), compared with the EAE animal models, the mean score was dramatically lower in the EAE mice transplanted with hADSCs (Fig. 2B). Taken together, these results showed that, transplantation of hADSCs could alleviate the body weight loss and neurological function impairment in the EAE mice.

### Effects of hADSC transplantation on histopathological changes in spinal cord of EAE mice

**Figure 3:**
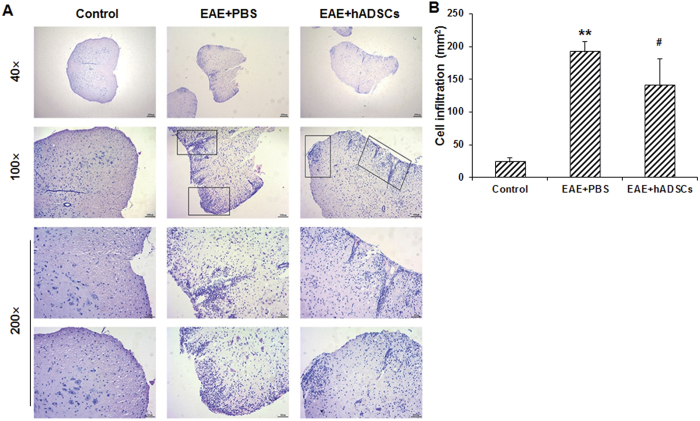
Effect of hADSC transplantation on inflammatory cell infiltration in the spinal cord of the EAE mice. (**A**) Inflammatory cell infiltration in the spinal cord of the mice in the control, EAE model (EAE + PBS), and hADSC transplantation (EAE + hADSCs) groups was detected with H&E staining (40×, 100×, and 200×, respectively). (**B**) Statistical analysis of inflammatory cell infiltration. Compared with the control group, **P < 0.01; compared with the EAE model group, ^#^*P* < 0.05.

To investigate the effects of hADSC transplantation on histopathological changes in the EAE mice, H&E and LFB staining was performed. Our results from the H&E staining showed that, in the control group, clear and regular spinal structure was observed, with no significant infiltration of inflammatory cells (Fig. 3). In the EAE model group, the inflammatory cells were significantly increased compared with the control group (*P* < 0.01), and inflammatory cell infiltration was even noted in the gray matter (Fig. 3). Moreover, obvious perivascular cuff was observed in the model group. However, in the hADSC transplantation group, the inflammatory cells were dramatically reduced compared with the EAE model group (*P* < 0.05), and the infiltration area was drastically decreased (Fig. 3).

**Figure 4:**
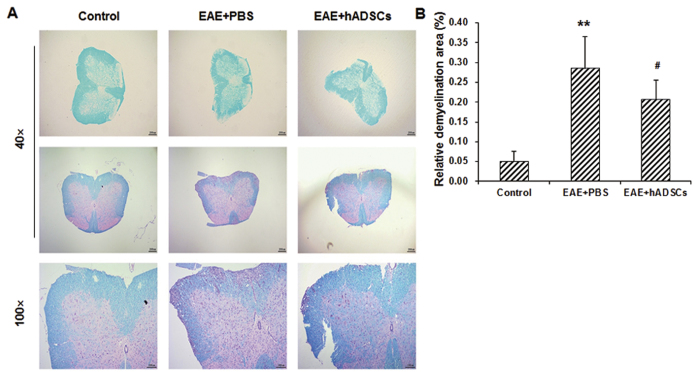
Effect of hADSC transplantation on demyelination in the EAE mice. (**A**) Demyelination in the mice of the control, EAE model (EAE + PBS), and hADSC transplantation (EAE + hADSCs) groups was detected with LFB staining (40×, 100×, and 200×, respectively). (**B**) Statistical analysis of relative demyelination area. Compared with the control group, **P < 0.01; compared with the EAE model group, ^#^*P* < 0.05.

Our results from the LFB staining showed that, compared with the control group, the blue-staining area in the peripheral region was significantly declined in the model group, indicating severe demyelination (the demyelination area was significantly elevated, *P* < 0.01), which was mainly located in the white matter and spinal cord surface (Fig. 4). Moreover, H&E staining also revealed a large number of inflammatory cells in the demyelination area. In the transplantation group, the pathological changes were dramatically alleviated, and the demyelination area was significantly decreased, compared with the EAE model group (*P* < 0.05) (Fig. 4). Taken together, these results suggest that, the transplantation of hADSCs could significantly alleviate the histopathological alterations in the EAE mice.

### Effects of hADSC transplantation on Th17 and Treg cells in spleen of EAE mice

**Figure 5:**
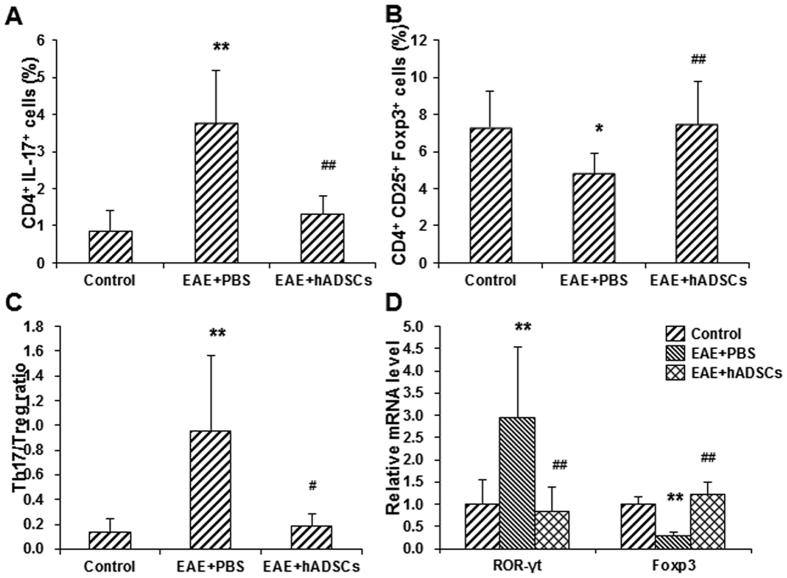
Effect of hADSC transplantation on the Th17 and Treg cells in the spleen of EAE mice. (**A**) Statistical analysis of the percentage of CD4^+^ IL-17A^+^ Th17 cells out of the total CD4^+^ T cells. (**B**) Statistical analysis of the percentage of CD4^+^ CD25^+^ Foxp3^+^ Treg cells out of the total CD4^+^ T cells. (**C**) Statistical analysis of the Th17/Treg ratio. (**D**) The mRNA expression levels of ROR-γt (Th17-specific marker) and Foxp3 (Treg-specific marker) were detected with real-time PCR. Compared with the control group, *P < 0.05, **P < 0.01; compared with the EAE model group, ^#^*P* < 0.05, ^##^*P* < 0.01.

To investigate the effects of hADSC transplantation on Th17 and Treg cells in the EAE mice, these cells were detected with flow cytometry. Our results showed that, compared with the control group, the percentage of the CD4^+^ IL-17A^+^ cells (i.e., Th17 cells) out of the CD4^+^ T cells was significantly elevated in the EAE model group (*P* < 0.01), which was then significantly declined by the transplantation of hADSCs (*P* < 0.01) (Fig. 5A). For the Treg cells, compared with the control group, the percentage of CD4^+^ CD25^+^ Foxp3^+^ cells (i.e., Treg cells) out of the CD4^+^ T cells was significantly declined in the model group (*P* < 0.05), which was then significantly increased in the hADSC transplantation group (*P* < 0.01) (Fig. 5B). Based on these results, compared with the control group, the Th17/Treg ratio was significantly increased in the EAE model group (*P* < 0.01), which was then significantly declined in the hADSC transplantation group (*P* < 0.05) (Fig. 5C).

The mRNA expression levels of Th17- and Treg-specific transcription factors (i.e., ROR-γt for Th17 cells, and Foxp3 for Treg cells) in the spleen tissue were detected with real-time PCR. Our results showed that, compared with the control group, the mRNA expression level of ROR-γt was significantly elevated in the spleen of the EAE mice (*P* < 0.01), which was significantly declined by the hADSC transplantation (*P* < 0.01) (Fig. 5D). On the other hand, compared with the control group, the mRNA expression level of Foxp3 was significantly decreased in the spleen of the EAE mice (*P* < 0.01), which was significantly increased in the hADSC transplantation group (*P* < 0.01) (Fig. 5D). These results were in line with the above findings. Taken together, these results suggest that, the transplantation of hADSCs could affect the Th17 and Treg cells, and consequently influence the Th17/Treg ratio, in the spleen of EAE mice.

### Effect of hADSC transplantation on pro-inflammatory cytokines in EAE mice

**Figure 6:**
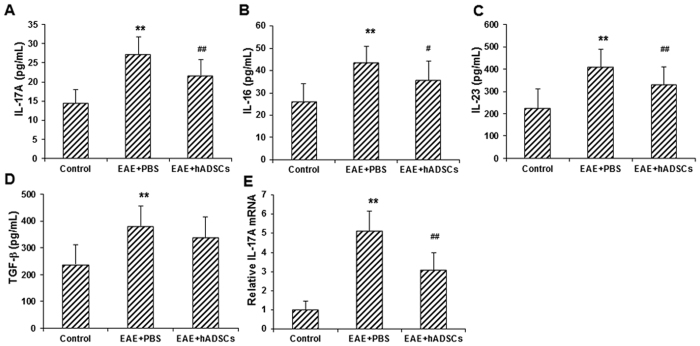
Effect of hADSC transplantation on the levels of pro-inflammatory cytokines in the EAE mice. (**A**–**D**) The serum levels of IL-17A (**A**), IL-6 (**B**), IL-23 (**C**), and TGF-β (**D**) in the control, EAE model, and hADSC transplantation groups were detected with flow cytometry. (**E**) The mRNA expression level of IL-17A in the spleen was detected with real-time PCR. Compared with the control group, **P < 0.01; compared with the EAE model group, ^#^*P* < 0.05, ^##^*P* < 0.01.

To investigate the effects of hADSC transplantation on the pro-inflammatory cytokines in the EAE mice, the serum contents of IL-17A, IL-6, and IL-23, as well as TGF-β, were determined with ELISA, and the mRNA expression level of IL-17A in the spleen tissue was detected with real-time PCR. Our results from ELISA showed that, compared with the control group, the serum contents of all these pro-inflammatory cytokines, IL-17A, IL-6, and IL-23, as well as TGF-β, were significantly elevated in the EAE model group (all *P* < 0.01) (Fig. 6A–D). However, the transplantation of hADSCs significantly declined the serum contents of these pro-inflammatory cytokines (*P* < 0.01 for IL-17A, IL-23, and TGF-β; and *P* < 0.05 for IL-6), while induced limited influence on the serum level of TGF-β (Fig. 6A–D). Similar results were obtained for the detection of IL-17A mRNA in the spleen. Compared with the control group, the mRNA expression level of IL-17A in the spleen was significantly elevated in the EAE model group (*P* < 0.01), which was significantly declined by the transplantation of hADSCs (*P* < 0.01) (Fig. 6E). Taken together, these results suggest that, the hADSC transplantation could significantly alleviate the elevation in pro-inflammatory cytokine levels in the EAE mice.

### Homing of PKH67-labeled hADSCs in EAE mice

**Figure 7:**
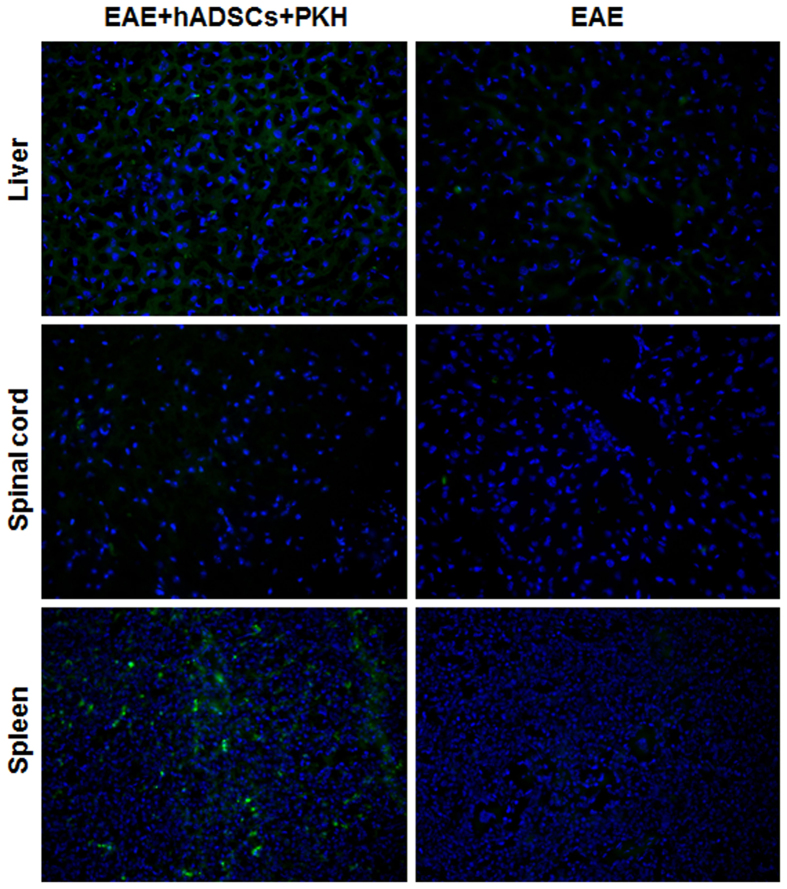
Homing of PKH67-labeled hADSCs in EAE mice. The hADSCs were labeled with PKH67 on day 14 of immunization, and tracked in the spleen, brain, and liver at day 8 after injection (400×).

In order to observe where the transplanted hADSCs migrated and exerted the immunoregulatory effects, these cells were labeled with PKH67 on day 14 of immunization, and tracked in the lymphoid organs, brain, and liver at day 8 after injection. As shown in Fig. 7, almost no PKH67-hADSCs were detected in the brain, and they were also rarely seen in the liver. However, a large number of PKH67-hADSCs were observed infiltrating the spleen.

## Discussion

In recent years, the researches on the treatment of MS have been focusing on the transplantation of stem cells, including hematopoietic stem cells^22^, BMSCs^23^, neural precursor cells (NPCs)^24^, and amniotic mesenchymal stem cells^25^,^26^. In the present study, hADSCs were isolated, cultured, and transplanted into the EAE mice. In 2001, Zuk *et al*.^27^ have shown that, the spindle cells isolated and cultured form human adipose tissue (i.e., hADSCs) are pluripotent stem cells, the same as BMSCs. Moreover, hADSCs have been characterized by the low immunogenicity, which would not lead to xenograft or allograft rejection^28^. In the present study, our results from flow cytometry showed that, in the isolated and cultured cells herein, CD29, CD44, CD73, and CD105 were highly expressed, while CD34 (hematopoietic stem cell surface marker) and CD 31 (endothelial progenitor molecular cell surface marker) were at low expression levels, with no expression of HLA-DR, which was in line with previous findings^23^. Therefore, these isolated, cultured, and purified hADSCs were confirmed by the immunogenic properties, which were suitable for the investigation in this study.

It has been accepted before that, the therapeutic effects of stem cells on neurological autoimmune diseases are based on the differentiation of these cells into neural cells and the ability to promote tissue regeneration. However, many studies have demonstrated that, it is not necessary to transplant BMSCs or NPCs directly into the CNS to treat EAE animals. These cells could exert beneficial effects via regulating peripheral immune responses, suppressing pathogenic processes, and releasing neuroprotective factors^24^,^29^,^30^. It has been reported that, BMSCs can induce the differentiation of peripheral Treg cells to exert protective effects on EAE animals^14^,^15^,^16^. Moreover, for the comparison of therapeutic effects between different MSCs, Mehdi *et al*.^31^ have shown that, under some circumstances, ADSCs could exert stronger immunomodulatory capacity than BMSCs. Based on these findings, in the present study, hADSCs were injected into the EAE mice via tail vein, and our results showed that, compared with the EAE model group, the neurological function score was significantly lower, and the inflammatory infiltration and demyelination in the CNS were significantly alleviated, in the hADSC transplantation group. Together with previous findings^18^, we suppose that hADSCs could exert protective effects on EAE mice, which might be associated with not only its regeneration capacity in the lesions, but also with its potent immunomodulatory effects.

To explore the mechanisms for the immune protective effects of hADSCs, Th17 and Treg cells in the spleen of EAE mice were first investigated. Our results showed that, compared with the control group, the Th17/Treg ratio was significantly elevated in the EAE mice, which could be declined by the transplantation of hADSCs. Th17 subset cells are the main effector cells mediating immune responses and demyelination in MS/EAE. On the other hand, the CD4^+^ CD25^+^ Foxp3^+^ Treg cells, as protective factors in the autoimmunity and tissue damaging process, are also targets for various therapeutic treatments. Due to the differentiation plasticity, Th17 and Treg cells could convert into each other under different cytokine environments. Therefore, the Th17/Treg ratio could partially reflect the peripheral immune imbalances in EAE. Our results showed that, compared with the control group, imbalanced Th17/Treg ratio occurred in the EAE mice, which could be alleviated by the transplantation of hADSCs.

In addition, our results also showed that, the serum levels of IL-17A, IL-6, IL-23, and TGF-β were significantly elevated in the EAE model group. IL-17A is a pro-inflammatory cytokine secreted by Th17 cells, which is also the major effector in Th17 cell-induced inflammation response. IL-6 is mainly secreted by the cells from the innate immune system, which plays an important role in the differentiation of CD4^+^ T cells into the Th17 cells. It has been shown that, mutually exclusive relationship exists in the induction and differentiation between the Th17 and Treg cells^32^. The pro-inflammatory cytokine IL-6 in the body would suppress the Foxp3^+^ Treg cells, and the inhibition of IL-6 would promote the development of these cells. Interestingly, IL-6 has been recently shown to be able to mediate the transition from the differentiated Foxp3^+^ Treg cells into the Th17 cells, while IL-17 secreted by the Th17 cells could promote the expression of IL-6, forming a potential positive feedback loop^33^. A previous study has demonstrated that, both IL-6 and IL-17 would be up-regulated in the MS/EAE models, even though at different time points^34^. Furthermore, IL-23 is another key factor in maintaining the proliferation of Th17 cells. It has been shown that, IL-23 would promote the accumulation of myelin-reactive T cells in the CNS, and mediate the secretion of activators like G-CSF^35^. ADSCs have also been shown to directly act on IL-23 receptor-related signaling pathway to inhibit the differentiation of Th17 cells in the patients with systemic lupus erythematosus. Our results showed that, the transplantation of hADSCs significantly down-regulated the serum levels of IL-17A, IL-6, and IL-23 in the EAE mice, indicating that the mechanisms of the protective effects of hADSCs might be associated with the regulation of the Th17 differentiation environment, forcing the Th17/Treg balance towards the Treg differentiation. Therefore, the secretion of IL-17A would be decreased, and the attack of peripheral immune cells to the myelin would be prevented. On the other hand, the Th17/Treg imbalance increases the Treg protective cells, enhancing the inhibition of the body’s immune responses. In line with these, our results showed that, compared with the EAE model group, the transplantation of hADSCs significantly down-regulated the mRNA expression level of ROR-γt (Th17-specific transcription factor), while up-regulated the Foxp3 mRNA expression level (Treg-specific transcription factor), in the EAE mice.

TGF-β is a key regulator of the differentiation of Th17 and Treg cells. Together with IL-10, TGF-β could induce the immature CD4^+^ T cells to differentiate into Treg cells, and the Treg cells could produce large amount of TGF-β. TGF-β has been shown to be able to exert anti-inflammation effects, and mediate the expression of Foxp3. However, a recent *in vitro* study demonstrates that, together with the synergistic stimulation of IL-6, TGF-β could promote the differentiation of Th17 cells. Based on these findings, TGF-β would promote the differentiation of both Th17 and Treg cells, which depends on the stimulation of IL-16. Our results showed that, the serum level of TGF-β was also elevated in the EAE mice, which might, together with the elevated IL-6 and IL-23 levels, form the pro-inflammatory environment to stimulate the differentiation of Th17 cells, contributing to the disease development. However, the transplantation of hADSCs significantly inhibited the secretion of IL-6 and IL-23 in the EAE mice, while induced limited influence on the serum TGF-β level. The changes would effectively inhibit the Th17 cells while promote the Treg cell differentiation, forcing the Th17/Treg balance to benefit the disease remission. In the present study, our *in vivo* results showed that, the transplantation of hADSCs could regulate the balance of the Th17/Treg axis to exert beneficial effects on EAE mice. Of course, further in-depth studies are still needed to elucidate the detailed mechanisms.

In conclusion, hADSCs were successfully isolated, purified, and identified, and the EAE mouse model was established by the active immunization with MOG35-55. The mice were transplanted with purified hADSCs. Our results showed that, the transplantation of hADSCs would significantly alleviate the neurological function and histological changes in these EAE mice, and reduce the inflammatory cell infiltration and demyelination in the CNS. The mechanisms of the protective effects of hADSCs might be associated with the regulation of the Th17/Treg balance axis. These findings might provide the evidence for the application of hADSC transplantation in the treatment of MS in clinic.

## Additional Information

**How to cite this article**: Li, J. *et al*. Therapeutic effects of human adipose tissue-derived stem cell (hADSC) transplantation on experimental autoimmune encephalomyelitis (EAE) mice. *Sci. Rep.* **7**, 42695; doi: 10.1038/srep42695 (2017).

**Publisher's note:** Springer Nature remains neutral with regard to jurisdictional claims in published maps and institutional affiliations.
